# Effects of three different probiotics of Tibetan sheep origin and their complex probiotics on intestinal damage, immunity, and immune signaling pathways of mice infected with *Clostridium perfringens* type C

**DOI:** 10.3389/fmicb.2023.1177232

**Published:** 2023-04-17

**Authors:** Xi He, Guisheng Ye, Shuqin Xu, Xiaohui Chen, Xiaolong He, Zifeng Gong

**Affiliations:** College of Agriculture and Animal Husbandry, Qinghai University, Xining, Qinghai, China

**Keywords:** probiotics, complex probiotics, *Clostridium perfringens*, small intestinal mechanical barrier, NF-κB signaling pathway, MAPK signaling pathway

## Abstract

Tibetan sheep have unique intestinal microorganisms in their intestines that are adapted to the highland alpine and anoxic environment. To further clarify the probiotic properties of Tibetan sheep-derived probiotics, we selected three Tibetan sheep-derived probiotic isolates (*Enterococcus faecalis* EF1-mh, *Bacillus subtilis* BS1-ql, and *Lactobacillus sakei* LS-ql) to investigate the protective mechanisms of monocultures and their complex strains against *Clostridium perfringens* type C infection in mice. We established a model of *C. perfringens* type C infection and used histology and molecular biology to analyze the effects and mechanisms of different probiotic treatments on mice after *C. perfringens* type C infection. After supplementation with either probiotics or complex probiotics, mice were improved in terms of weight reduction and reduced the levels of cytokines in serum and increased the levels of intestinal sIgA, and supplementation with complex probiotics was effective. In addition, both probiotic and complex probiotic supplementation effectively improved the damage of intestinal mucosa and spleen tissue. The relative expressions of *Muc 2*, *Claudin-1*, and *Occludin* genes were increased in the ileum. The three probiotics and the compound probiotics treatment significantly reduced the relative mRNA expression of toll-like/MyD88/NF-κB/MAPK. The effect of probiotic treatment was similar to the results of engramycin treatment, but the effect of engramycin treatment on intestinal sIgA was not significant. Our results clarify the immunomodulatory effects of the three probiotic isolates and the complex probiotics on *C. perfringens* infection, and the repair of the intestinal mucosal barrier.

## 1. Introduction

Tibetan sheep is one of the original breeds in China (Niu et al., 2017), mainly distributed in the Qinghai-Tibet Plateau (above 3,500 m in altitude), and has long been adapted to the alpine and high-pressure environment of the plateau. Past studies have demonstrated structural differences in the intestinal flora of Tibetans living at high altitudes in China compared to other regional ethnic groups (Zhang et al., 2015). The same has been demonstrated for yaks, an ancient organism of high altitude, where probiotic strains isolated from yaks improved growth performance in mice, as well as inflammatory and immune-related indices (Li et al., 2019). Therefore, specific intestinal bacteria may also be present in Tibetan sheep and fewer studies have been reported on intestinal probiotic strains in Tibetan sheep.

Probiotics are defined as living microorganisms that when used in sufficient amounts can provide beneficial effects on host health (Hill et al., 2014). In livestock farming, probiotics are often used to promote the absorption of nutrients by the organism (Singh et al., 2019), improve growth performance and meat quality (Abou-Kassem et al., 2021). More importantly, probiotics can protect the intestinal mucosal barrier (Liu et al., 2016), and improve the immunity of the organism (Xie et al., 2019). In addition, probiotics produce antimicrobial substances; compete with pathogens for binding sites in intestinal epithelial cells; and regulate the brain-gut axis by producing neurotransmitters (Yan and Polk, 2020; Gabanyi et al., 2022). When the organism is infected with pathogenic bacteria, immune responses are generated mainly through toll like receptor (TLR) that recognize pathogenic bacteria-related molecules and induce activation of nuclear factor kappa-B (NF-κB), mitogen-activated Protein Kinases (MAPKs), and activating protein-1 (AP-1) signaling pathways (Yang et al., 2015; Yu et al., 2022). Probiotics have been shown to inhibit NF-κB signaling pathway and reduce the level of inflammation in the body, exerting beneficial effects (Wu et al., 2022). And probiotics can significantly reduce serum inflammatory cytokines IL-1β, IL-6, and TNF-α (Liang et al., 2019; Jin and Chen, 2020; Mehrabadi and Sadr, 2020; Wang T. et al., 2020). In organismal intestinal injury, the expression of intestinal tight junction factors Occludin, Claudin-1, and Zonulin-1 can be increased by the addition of probiotics to reduce intestinal injury (Deng et al., 2021; Guo et al., 2021; Panpetch et al., 2021). Currently, the common probiotics mainly include *Lactobacillus*, *Bacillus*, *Bifidobacterium*, and some Gram-positive *cocci* (Konstantinov et al., 2008).

Different probiotics sometimes show great variation and may differ in their inhibitory effects on pathogenic bacteria. Compared to individual probiotics, there is a synergistic effect within the complex probiotics (Lin et al., 2017; Zhang et al., 2022). Complex probiotics are a mixture of two and more probiotics in a certain ratio. The intestinal environment is complex, with different genera of bacteria distributed, and each strain has different unique effects on the health of the host, and the complex probiotic can integrate each probiotic with different effects, and the complex probiotic can exert better probiotic effects than a single strain. In a therapeutic study of ulcerative colitis, four different probiotics were found to differ in the regulation of intestinal immunity and barrier function, and when mixed they significantly enhanced the expression of IL-10, the intestinal barrier function (Wang Y. et al., 2020). After the addition of three different *Lactobacillus plantarum* and their mixtures to healthy rats, respectively, the mixed probiotics modulated the diversity of intestinal microorganisms in rats more significantly than the single probiotics (He et al., 2021). The combination of *L. plantarum* and *Lactobacillus curvatus* was more effective in regulating hepatic lipid metabolism and inhibiting diet-induced obesity (Yoo et al., 2013). Therefore, the maximum beneficial effect in the host is ensured by selecting the appropriate probiotic strains or combining them in a certain ratio to become a complex probiotic.

*Clostridium perfringens* is a common human-animal conditional pathogen and can cause necrotizing necrotic enteritis, enterotoxaemia (Uzal et al., 2014), and gas gangrene in livestock and poultry. *C. perfringens* can produce more than ten types of toxins. Among them, alpha toxins and beta toxins can cause damage to the intestinal villus epithelium and endothelial cells, destroy the structural integrity of the cell membrane, and change the permeability of the intestinal wall to induce enteritis; after mucosal damage some toxins are absorbed into the blood circulation and affect other organs distal to the host (Uzal and McClane, 2011; Hussain et al., 2022). In past livestock farming, *C. perfringens* infections were often prevented by adding antibiotics to the feed. With the gradual ban of antibiotics in livestock farming, the farming industry has started to look for antibiotic alternatives to prevent, alleviate or control *C. perfringens* infections. Currently available antibiotic alternatives mainly include probiotics, prebiotics, synbiotics, organic acids, enzymes, etc. (Matsushita and Okabe, 2001). Among them, probiotics are widely studied as natural, safe, and effective additives.

In our previous study, we isolated and identified three strains with probiotic potential from healthy Euler-type Tibetan sheep, namely *Enterococcus faecalis* EF1-mh (Xiao-hui Chen et al., 2021), *Bacillus subtilis* BS1-ql (Shu-qin Xu et al., 2022), and *Lactobacillus sakei* LS-ql (Xiang-zhao Ma et al., 2021). These three strains of probiotics have a wide range of beneficial properties, including acid and alkaline resistance, non-drug resistance, and strong inhibition of common pathogenic bacteria. The purpose of this study was to clarify the effects of three Tibetan sheep-derived probiotics, alone or in combination, on tissue damage and inflammatory responses in *C. perfringens*-infected mice, and to understand the protective mechanisms of probiotics on the host.

## 2. Materials and methods

### 2.1. Animal ethics statement

The animal experiments were approved by the ethics committee of Qinghai University (SL-2022010). Every effort is made to minimize animal suffering, and all animal experimental procedures are performed in accordance with the guidelines of *The Animal Welfare Act*.

### 2.2. Animals and diet

The clean-grade Kunming mice (4–5 weeks old) used in the experiments were purchased from the Himalayan Experimental Animal Center in Qinghai Province. Mice were fed at 23–25°C and 50% humidity under a 12 h light/dark cycle with free access to food and water.

### 2.3. Preparation of probiotics and *C. perfringens*

*Enterococcus faecalis* EF1-mh, *Bacillus subtilis* BS1-ql, and *Lactobacillus sakei* LS-ql were isolated and identified and preserved by the Qinghai-Tibet Plateau Animal Disease Research Laboratory, Qinghai University. *C. perfringens* type C was purchased from the China Veterinary Microbial Strain Collection Management Center (CVCC75).

The three strains of probiotics were resuscitated and inoculated in Luria-Bertani (LB) culture medium overnight, and the overnight cultures were collected and washed with sterilized phosphate buffered saline (PBS) (pH 7.4) and resuspended to adjust the concentration to 10^8^ CFU/ml. The three strains of probiotics with adjusted concentrations were mixed 1:1:1 to make a complex probiotic. *C. perfringens* type C was transferred to liquid thioglycolate medium at 1% before infection, and the overnight cultures were washed and resuspended with sterilized PBS (pH 7.4) to adjust the concentration to 10^9^ CFU/ml.

### 2.4. Experimental design and sample collection

The mice were weighed after 1 week of adaptive feeding and randomly divided into eight groups of 10 mice each. Except for the negative control group, all groups of mice were pretreated with neomycin (0.2 mg/tail) by gavage 24 h before the start of the experiment. The groups were as follows: (NC) Negative control: PBS (0.2 ml/tail, administered daily for 13 days by gavage); (CPM) *C. perfringens* infection model: *C. perfringens* (2 × 10^8^ CFU/tail, administered daily for 8 days by gavage); (CPC) *C. perfringens* infection positive control: *C. perfringens* (2 × 10^8^ CFU/tail, administered daily for 8 days by gavage), PBS followed (0.2 mL/tail, administered daily for 5 days by gavage); (EDC) *C. perfringens* + Engramycin: *C. perfringens* (2 × 10^8^ CFU/tail, administered daily for 8 days by gavage), engramycin followed (1 mg/tail, administered daily for 5 days by gavage); (EF) *C. perfringens* + E. faecalis EF1-mh: *C. perfringens* (2 × 10^8^ CFU/tail, administered daily for 8 days by gavage), EF1-mh followed (2 × 10^7^ CFU/tail, administered daily for 5 days by gavage); (BS) *C. perfringens* + B. subtilis BS1-ql: *C. perfringens* (2 × 10^8^ CFU/tail, administered daily for 8 days by gavage), BS1-ql followed (2 × 10^7^ CFU/tail, administered daily for 5 days by gavage); (LS) *C. perfringens* + L. sake SK-ql: *C. perfringens* (2 × 10^8^ CFU/tail, administered daily for 8 days by gavage), SK-ql followed (2 × 10^7^ CFU/tail, administered daily for 5 days by gavage); (MC) *C. perfringens* + Multistrain Combination: *C. perfringens* (2 × 10^8^ CFU/tail, administered daily for 8 days by gavage), multistrain combination followed (2 × 10^7^ CFU/tail, administered daily for 5 days by gavage). In order to maintain the activity of the strain, the bacterial solution used in the experiment was prepared daily and gavaged regularly, while the weight change and mental status of the mice were recorded daily.

### 2.5. Mouse sample preparation

The mice were weighed after fasting for 12 h before the end of the test, and blood samples were collected from mice in NC, CPM, CPC, EDC, EF, BS, LS, and MC groups by removing their eyes, and the blood was naturally rested at 37°C for 2 h and then centrifuged at 2,500 r/min for 20 min to prepare serum, and stored at −20°C. After blood collection, the mice were dislocated from the cervical vertebrae, and the spleen was collected aseptically for weighing and calculating the spleen organ index. After the spleen was weighed, part of it was put into 4% paraformaldehyde fixative, and the remaining visceral organs were rapidly frozen in liquid nitrogen for preservation. The duodenum, jejunum and ileum were removed, and part of them were put into the fixing solution, and the remaining part was rapidly frozen in liquid nitrogen and then put into −80°C refrigerator for storage.


SpleenIndex(%)=Spleenweight(g)/Bodyweight(g)×100%

### 2.6. Detection of immunoglobulins and cytokines in serum and intestine

The concentrations of cytokines and immunoglobulins in serum were determined using mouse-specific IL-1β, IL-2, IL-4, IL-6, TNF-α, IFN-γ, and IgG quantification kits (Kit code: MB-2776A, MB-2903A, MB-3400A, MB-2899A, MB-2868A, MB-2918A, MB-2793A. Jiangsu Enzyme Labeling, China). The intestinal contents of mice were collected and centrifuged with PBS resuspension, and the supernatant was retained. The amount of intestinal sIgA was determined using a mouse-specific ELISA (Enzyme Linked Immunosorbent Assay, ELISA) kit (Kit code: MB-3166B. Jiangsu Enzyme Labeling, China). The absorbance of each well at a wavelength of 450 nm was measured using a microplate reader.

### 2.7. Histological observation of the small intestine and spleen of mice

Duodenal, jejunum, ileum and spleen tissues from mice were soaked in 4% paraformaldehyde for 1 week and used to make tissue sections. The sections were observed histologically by optical microscope (OLYMPUS BX51, Japan). The degree of small intestinal inflammation was also determined, and the villus height, crypt depth and villus height/crypt depth of small intestinal tissues were measured, and the mean values were calculated and statistically analyzed.

### 2.8. Transmission electron microscope observation of tight junctions in mice ileum

Mice ileal tissues were collected aseptically, rinsed with pre-chilled PBS and placed in 2.5% glutaraldehyde fixative for making sections, and the sections were observed by transmission electron microscopy (HITACHI HT7700, Japan) for the level of tight junctions and adhesive junctions of ileal epithelial cells.

### 2.9. Determination of relative gene mRNA expression by quantitative real-time PCR

Total RNA was extracted from ileal tissue using a tissue RNA extraction kit (Kit code: RN28, Aidlab, China), and the concentration and purity of RNA were measured using a micro-nucleic acid protein concentration assay (Gene Company Limited, China). Reverse transcription of RNA to cDNA according to cDNA synthesis kit (Kit code: RR047A, TaKaRa, China) instructions.

The qRT-PCR (Quantitative Real-time Polymerase Chain Reaction, qRT-PCR) reactions were performed using SYBR premix (Kit code: RR820A, TaKaRa, China) in a LightCycler^®^ 96 real-time fluorescent quantitative PCR system (Roche, Germany). The house-keeping gene β-*actin* was used as a reference gene. The primer sequences and internal reference gene sequences used for real-time fluorescent quantitative PCR are shown in Supplementary Table 1. The qRT-PCR results were analyzed using the 2^–ΔΔ*Ct*^ method for data analysis (Gadde et al., 2017).

### 2.10. Statistical analysis

Results were expressed as mean ± SEM (Standard Error of Mean) for variables with normal distribution. Statistical analyses between groups were performed using the analysis of variance (ANOVA) followed by the multiple comparison test of Bonferroni. Differences were considered statistically significant when *P* < 0.05. The analyses were done using GraphPad PRISM^®^ Software, version 6.0 (Graph Pad, La Jolla, CA, USA).

## 3. Results

### 3.1. Clinical performance, body weight change and spleen index of mice after different probiotic interventions

**FIGURE 1:**
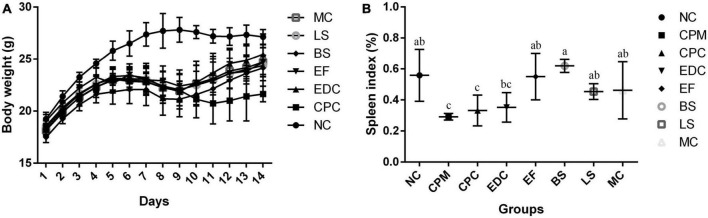
**(A)** Body weight changes of mice during the experiment (*n* = 8); **(B)** Spleen index plot (*n* = 6). a–c Means with different superscripts within a row were significantly different (*P* < 0.05). The CPM group of mice was only used to verify the success of the model, therefore body weight is not listed.

In order to investigate the intervention effects of three probiotics and a combination of probiotics on *C. perfringens* type C infected mice, the mental status and body weight changes of different groups of mice were firstly monitored. In the NC group, the mice showed normal mental status and food intake, and no significant decrease in body weight. In the other groups, mice in the first to fifth days of *C. perfringens* type C attack had reduced diet and water intake, reduced activity, and slow weight gain compared with the NC group; from the fifth to the eighth days, mice were depressed, activity was significantly reduced, coat was slightly ragged, diet and water intake were reduced, and weight loss began. On day 9, mice in the CPC group were depressed, had less food and water, less activity, and were grouped together, and their body weight continued to decrease, but on day 12, their body weight started to increase. From day 10 onwards, the EDC, EF, BS, LS, and MC groups showed improvements in mental performance and weight gain compared to the CPC group. In contrast, the CPC group was still losing weight in the absence of *C. perfringens* infection and slowly regained weight gain after day 12 (Figure 1A).

The spleen index of different groups of mice were calculated. Spleen index in the CPM group were significantly lower than those in the NC group. Spleen index in the CPC group were not significantly different compared to the CPM group, but showed an increasing trend. Spleen index in the EDC group were not significantly different compared to the CPC group, but also showed an increasing trend. The spleen index was significantly higher in the EF, BS, LS, and MC groups compared to the CPC group (Figure 1B).

### 3.2. Morphology of the small intestine of mice in different treatment groups

**FIGURE 2:**
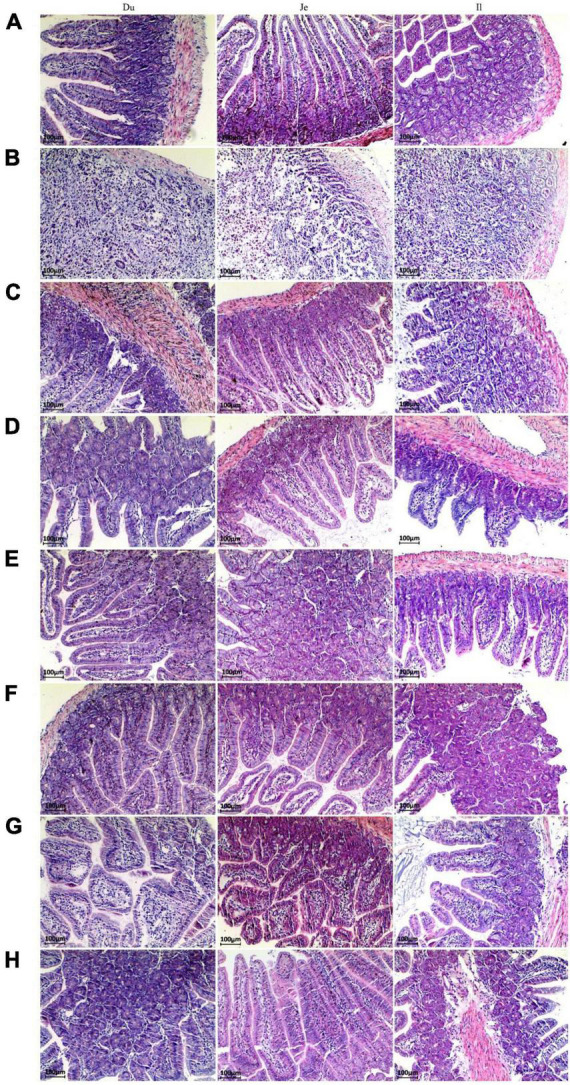
Paraffin sections of the small intestine of mice after different treatments. Morphological images of small intestine are shown at 40× magnification. Panels **(A–H)** represent NC, CPM, CPC, EDC, EF, BS, LS, MC group. Du: Duodenum; Je: Jejunum; Il: Ileum.

The pathological changes of small intestinal segments of mice after different treatments were investigated by morphology. The small intestinal villi in the NC group were structurally intact with clear tissue boundaries (Figure 2A). After infection with *C. perfringens* type C, the pathological changes in the small intestinal segment of the CPM group were obvious, with necrosis of the intestinal mucosa, loss of epithelium, necrosis of the lamina propria, and inconspicuous histological boundaries in the duodenum, jejunum, and ileum, accompanied by a large number of inflammatory cell infiltrations (Figure 2B). The intestinal villi of the duodenum, jejunum and ileum segments of the CPC group were destroyed, the villi epithelium was shed, and the ileum villi were severely broken. The mucosa of the jejunum and ileum appears congested (Figure 2C). After treatment with engramycin (EDC group) and probiotic (EF, BS, LS, and MC groups) intervention, the intestinal villi in each section of the small intestine were intact, and a small amount of inflammatory cell infiltration was seen in the mucosa (Figures 2D–H). Among them, the ileum mucosa was still congested, but the symptoms were less severe than those in the CPC group.

**FIGURE 3:**
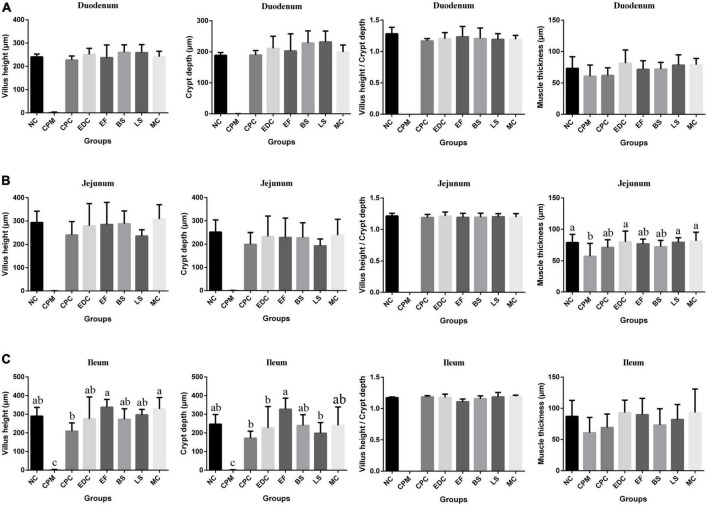
Morphological changes in the small intestine of mice after different treatments (*n* = 6). **(A)** Duodenum; **(B)** Jejunum; **(C)** Ileum. Data represent mean values ± SEM. a-c Means with different superscripts within a row were significantly different (*P* < 0.05).

Compared with the NC group, the CPM group had severe small intestinal damage and reduced muscle thickness in all segments of the small intestine after the establishment of the *C. perfringens* infection model (Figure 3). Compared with the CPM group, the length of intestinal villus height, crypt depth and muscle thickness of each segment of the small intestine in the CPC group were significantly improved, but still lower than those in the NC group. Among them, the villi length and crypt depth of ileum in the CPC group were still lower. Compared with the CPC group, the villus height, crypt depth, villus height/crypt depth and muscle thickness of all segments of the small intestine in the different treatment groups (EDC, EF, BS, LS, and MC groups) showed different degrees of increase, and the increase was significant in the ileum.

### 3.3. Effect of different probiotic interventions on spleen damage caused by *C. perfringens* type C infection in mice

**FIGURE 4:**
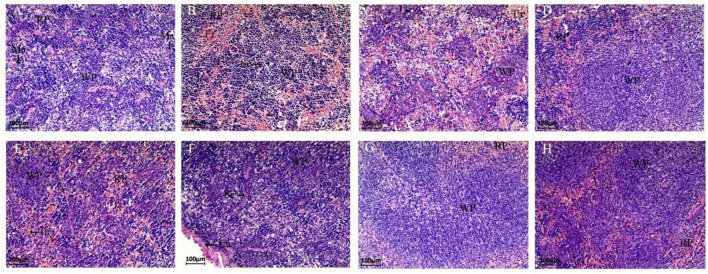
Paraffin sections of mouse spleen tissue after different treatments. Morphological images of the spleen are shown at 40 × magnification. Panels **(A–H)** represent NC, CPM, CPC, EDC, EF, BS, LS, MC group. Ca: Capsule; RP: Red pulp; WP: White pulp; Ma: Macrophages; Se: Septum; Er: Erythrocyte.

The spleen tissues of different groups of mice were observed by paraffin sections for damage (Figure 4). The spleen tissue of the NC group could be clearly distinguished from the red pulp, white pulp and macrophages under the microscope. In the CPM group, more hemorrhagic areas were seen in both the red pulp and white pulp, filled with erythrocytes and lymphocytes, etc. Also, the red pulp intrinsic cell component was reduced in this group. The white pulp of the CPC group was reduced, but a large number of erythrocytes were still visible, and the lymphoid tissue was distributed in islands, and some cells were necrotic Disintegration and diffuse necrosis were observed. Compared with the CPM group, the tissue cell necrosis was more severe in the CPC group, and the white pulp structure was not obvious. Compared with the CPC group, the EDC group showed a significant decrease in erythrocytes, and the red pulp and white pulp were clearly visible, basically restoring the normal morphological structure. The different probiotic-treated groups (EDC, EF, BS, LS, and MC groups) showed different degrees of improvement compared with the CPC group, and the erythrocytes were significantly reduced. Among them, the LS and MC groups showed more obvious improvement, and the white pulp volume increased and gradually normalized.

### 3.4. Effect of different probiotic interventions on cytokines and tight junctions in the ileal mucosa of *C. perfringens* infected mice

**FIGURE 5:**
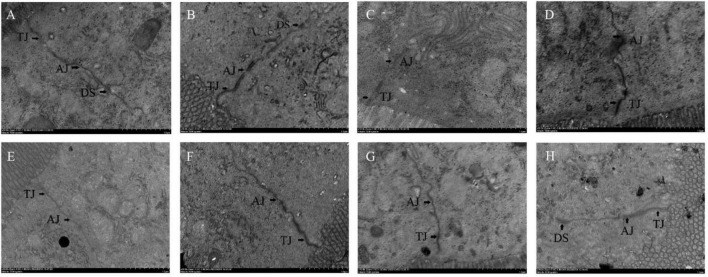
Transmission electron micrograph of mouse ileal tissue. Panels **(A–H)** represents NC, CPM, CPC, EDC, EF, BS, LS, MC group. TJ: Tight Junction; AJ: Adhesive Junction; DS: Desmosomes.

The effects of different probiotics on the intestinal barrier of mice were analyzed. The ileal mucosa of NC group mice was clearly visible under transmission electron microscope with tight intercellular junctions, and from the top of the cells downward, tight junctions, adhesive junctions and desmosomes could be clearly seen in turn, with no gaps and no loosening throughout (Figure 5A). Compared with the NC group, the cell gap in the CPM group did not change significantly, and the tight junctions and adherent junctions were slightly loosened (Figure 5B). Compared with the NC group, the CPC group had loose intercellular junctions, widened cell gaps at adhesive junctions, loose adhesive junctions, and blurred cytoskeletal fibers (Figure 5C). The ileum of mice with different interventions (EDC, EF, BS, LS and MC groups) were seen to have tight intercellular junctions at the upper level of most cells, with tight junctions, adhesive junctions and desmosomes structures intact (Figures 5D–H). Loose localized cellular junctions were seen in the middle and lower levels of some cells in the intervention group, but were lessened compared to the CPC group.

**FIGURE 6:**
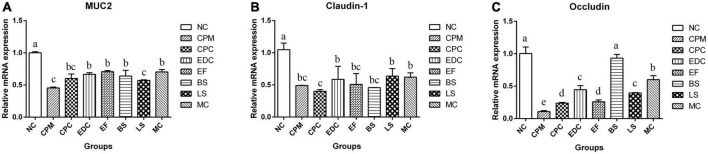
Changes in mucins and tight junction proteins after different treatments (*n* = 6). **(A–C)** The relative expression levels of *Muc 2*, *Claudin-1* and *Occludin* genes in the ileum were measured by qRT-PCR. Data represent mean values ± SEM. a-c Different letters indicate significant differences (*P* < 0.05).

The mRNA expression of mouse ileal mucin Muc2 (Mucoprotein2) and tight junction protein Claudin-1, Occludin were further investigated (Figure 6). The mRNA expressions of Muc2, Claudin-1 and Occludin were significantly lower in the CPM and CPC groups compared with the NC group. Meanwhile, the expression of Muc2 and Occludin in the CPC group was slightly higher than that in the CPM group. gene expression levels of Muc2 and Claudin-1 were restored in the EDC, EF, BS, LS, and MC groups. However, the expression of the tight junction protein Occludin was significantly restored only in the BS and MC groups, and was most significant in the BS group. These results suggest that the three probiotics have different effects on the regulation of mucosal barrier function. The combination treatment of the three probiotics was more effective in improving intestinal damage compared to single strains, and there was some synergistic effect among the three strains.

### 3.5. Different probiotic interventions improve the inflammatory response to *C. perfringens* type C infection in mice

**FIGURE 7:**
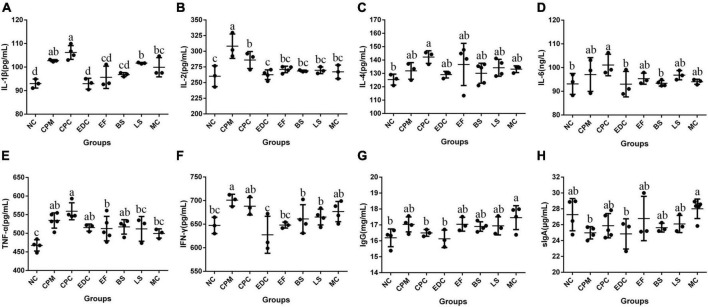
Cytokine and immunoglobulin levels after different treatments (*n* = 6). **(A–H)** Protein content of serum IL-1β, IL-2, IL-4, IL-6, TNF-α, IFN-γ, IgG, and intestine sIgA in mice. a-c means those with different letters are significantly different (*P* < 0.05).

Immunoglobulin IgG and cytokines IL-1β, IL-2, IL-4, IL-6, TNF-α, IFN-γ, and intestinal sIgA content in serum were analyzed (Figure 7). Compared with the NC group, IL-1β, IL-2, IL-4, IL-6, TNF-α, IFN-γ, and IgG contents were increased in the CPM group, with the most significant changes in the growth of IL-1β, IL-2, TNF-α, and IFN-γ. And the sIgA content was decreased in the CPM group. Compared with the CPM group, the serum IL-2 and IgG levels decreased in the CPC group, and the changes of other cytokine levels were not significant. The levels of IL-1β, IL-2, IL-6, TNF-α, and IFN-γ in the EDC, EF, BS, LS, and MC groups showed a decrease compared with the CPC group. Differently, the sIgA content of EDC also showed a decrease, while the IgG and sIgA contents were increased in the EF, BS, LS and MC groups. Compared to the single probiotic treatment, the complex probiotic (MC group) was more pronounced in its effect on regulating IgG and sIgA content. Probiotic treatment (EF, BS, LS, and MC groups) increased IgG and sIgA levels compared to engramycin treatment (EDC group), with the increase being more pronounced in the combined probiotic group.

### 3.6. Effects of different probiotic treatments on gene expression of TLR2/NF-κB and TLR2/MAPK signaling pathways

**FIGURE 8:**
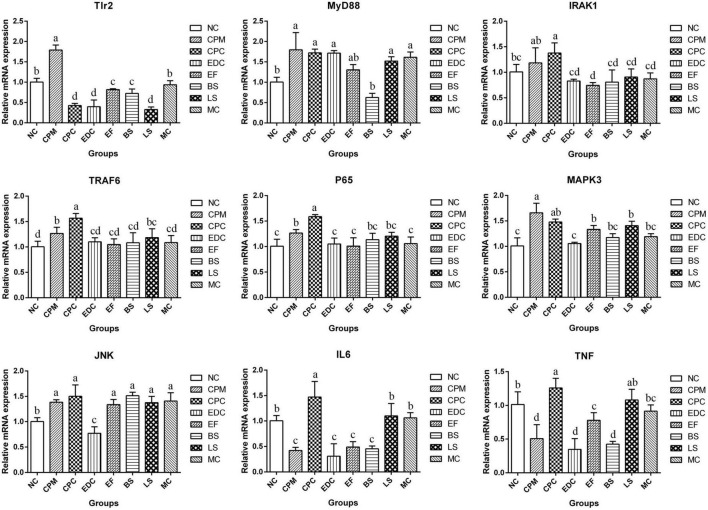
Expression of TLR2/NF-κB and TLR2/MAPK signaling pathway related genes in ileum after different treatments (*n* = 6). Data represent mean values ± SEM. a, b Means with different superscripts within a row were significantly different (*P* < 0.05).

To evaluate the potential mechanisms of different probiotic treatments on immune responses and inflammatory symptoms in *C. perfringens* type C infection mice, the relative expression of downstream target genes of TLRs signaling pathway was further determined by qRT-PCR (Figure 8). The expression of Tlr2, MyD88 (Myeloid Differentiation Factor 88), IRAK1 (Interleukin 1 Receptor Associated Kinase 1), TRAF6 (Tumor necrosis factor receptor-associated factor 6), P65, MAPK3 (Mitogen-Activated Protein Kinase 3), and JNK (c-Jun N-terminal kinase) mRNAs were significantly increased in the CPM group compared with the NC group. Compared with the CPM group, Tlr2 mRNA expression was significantly decreased in the CPC group; MyD88 and MAPK3 mRNA expression did not change significantly and showed a decrease; IRAK1, TRAF6, P65 and JNK mRNA expression increased, among which TRAF6 and P65 mRNA expression were significantly increased. Compared with CPC group, Tlr2 mRNA expression was significantly increased in EF, BS and MC groups; MyD88 mRNA expression was significantly decreased in BS group; IRAK1, TRAF6 and P65 mRNA expression were significantly decreased in EDC, EF, BS, LS and MC groups. MAPK3 and JNK mRNA expression were significantly decreased in EDC group. IL-6 and TNF-α mRNA expression were reduced by different probiotic treatments, and there were significant differences between the groups. The different probiotic treatments (EF, BS, LS, and MC groups) had similar levels of regulation of P65 mRNA expression in NF-κB signaling pathway compared with antibiotic treatment (EDC group); the same trend of regulation of MAPK3 and JNK mRNA expression in MAPK signaling pathway still existed, and the regulation effect was more significant in EDC group. The probiotic intervention groups (EF, BS, LS, and MC groups) had the same trend of regulation of IL-6 and TNF-α mRNA expression compared with the EDC group, and there were differences in the effects. The effect of compound probiotics on TLR2/NF-κB and TLR2/MAPK signaling pathway-related genes was more pronounced compared with that of single probiotics.

## 4. Discussion

Currently, probiotics are considered to be the most promising antibiotic alternative for fighting intestinal pathogenic infections, and the vast majority of probiotics have been shown to inhibit common pathogenic bacteria in the gastrointestinal tract by different means (Shi et al., 2019; Mendonça et al., 2022; Masset et al., 2023). *E. faecalis* EF1-mh, *B. subtilis* BS1-ql, and *L. sakei* LS-ql used in this study all have the ability to inhibit the growth of *C. perfringens*. In this study, the effects of three probiotics (*E. faecalis* EF1-mh, *B. subtilis* BS1-ql, *L. sakei* LS-ql) and their complex probiotics on intestinal morphological structure and immune function in mice were analyzed by *C. perfringens* type C infection mouse model. The results showed that the three probiotics and the complex probiotics could improve the intestinal inflammation and injury caused by *C. perfringens* bacteria to different degrees, and regulate the expression of TLR2, MyD88, IRAK1, TRAF6, P65, MAPK3, and JNK in ileum. The effect of the complex probiotics was more significant in improving intestinal morphology and modulating some immune factors.

Probiotics, as new feed additives, can improve growth performance and health of animals when used (Norris et al., 2017; Samolińska et al., 2018). Probiotics supplemented in lamb diets can improve growth performance by promoting the fermentation of rumen proteins (Kazemi et al., 2019). Probiotics of the Bacillus Cohn improve growth, health and production indicators in diseased broilers (Chen et al., 2021). In the present study, treatment of mice infected with *C. perfringens* type C with probiotic intervention and engramycin intervention improved the mental status and food intake of the mice, and they showed the same trend of weight gain. This suggests that the addition of probiotics can achieve the same effect as the use of antibiotics in terms of improving weight loss. However, Wang et al. (Ogbuewu et al., 2022) added complex probiotics to calf diets had no effect on body weight and daily weight gain. This may be related to the type and concentration of probiotics used and the way they were added. On the other hand, this study was conducted under *C. perfringens* infection conditions, and the probiotics improved the body’s absorption of nutrients and improved weight loss due to disease by regulating the intestinal environment and repairing intestinal damage. In addition, spleen index was significantly reduced in mice infected with *C. perfringens*. Enramycin treatment had little effect on the spleen index, but probiotic intervention significantly improved the spleen index, with *B. subtilis* BS1-ql having the most significant effect. Microscopic observation revealed that there were more hemorrhagic areas in the red pulp and white pulp of the spleen of mice infected with *C. perfringens* 8 days after infection, and the spleen tissue necrosis was more severe with diffuse necrosis 15 days after infection. Both engramycin and probiotic treatments were effective in alleviating infection-induced spleen damage, while the number of erythrocytes in the tissues decreased. Among them, *L. sakei* LS-ql and complex probiotics were more effective in improving spleen injury. The above results suggest that the aspects of action differed after different probiotic interventions.

Probiotics can effectively protect the intestinal mucosa of the body and improve the intestinal barrier function. *C. perfringens* type C infection occurs primarily in the small intestine. When *C. perfringens* type C proliferates in the host intestine, it forms transmembrane pores in susceptible cells of the host by producing the pore-forming toxin CPB. These pores cause K^+^ efflux and Ca^2+^, Na^+^, and Cl^–^ influx, leading to cell swelling and cell necrosis, causing intestinal villi breakage, necrosis, and autolysis (Uzal et al., 2014). Microscopic examination of mice inoculated with *C. perfringens* type C for 12 h showed atrophy of the small intestinal villi, dilatation of the intestinal lumen, severe weakening of the mucosa and blunting of the villi, and flattening of the short villi could be seen after 24 h (Uzal et al., 2009). Vaccination of goats with a wild strain of *C. perfringens* type C showed severe blunting of the villi, diffuse necrosis of the mucosa and submucosa, complete loss of the villi and crypt epithelium, and necrosis of the lamina propria (Garcia et al., 2012). In a rabbit ileal infection model, *C. perfringens* CN3685-treated intestine showed severe diffuse necrotizing enterocolitis, including epithelial necrosis and loss, complete loss of absorptive cells along the villi, coagulative necrosis of the lamina propria, almost complete loss of villi, mucosal hemorrhage, and diffuse neutrophil infiltration of the mucosa and submucosa (Sayeed et al., 2008). In this study, mice infected with *C. perfringens* type C for 8 days (CPM group) also showed mucosal necrosis, epithelial loss, blunting of villi, and inconspicuous histological boundaries in the small intestine, accompanied by a large infiltration of inflammatory cells. These phenomena were improved after treatment with probiotic intervention. All three probiotics and the complex probiotic were effective in improving small intestinal damage caused by *C. perfringens* type C infection, reducing the infiltration of inflammatory cells in small intestinal tissues and restoring the intestinal wall structure. The results of this study also showed that different probiotic and combination probiotic treatments increased villus height, crypt depth, villus height/crypt depth and muscle thickness in the small intestine and promoted post-infection recovery. In contrast, the recovery of ileum morphological structure was more significant with the complex probiotics and was higher than in the NC group. This part of the results is consistent with the results of previous studies. In a healthy population, the use of probiotics can reduce systemic inflammatory responses (Wang et al., 2022). In broilers infected with *C. perfringens*, the addition of *Bacillus subtilis* was effective in improving necrotizing intestinal lesions and regulating intestinal health (Li et al., 2017; Stene et al., 2022). *Lactobacillus casei* was effective in suppressing Salmonella induced intestinal inflammation in chicks and improving intestinal mucosal immunity (Cheng et al., 2018). In DSS (Dextran Sulfate Sodium Salt) induced colitis, the use of complex probiotics was more effective than single probiotics in preventing intestinal inflammation and injury (Deng et al., 2021). *Bacillus subtilis* PB6 significantly increased intestinal villus height and villus height to crypt depth ratio in broiler chickens with necrotizing enteritis (Ananthakrishnan et al., 2018). The ratio of villus height to crypt depth is considered to be the most important parameter for intestinal health and recovery from infection. *Clostridium butyricum* and *E. faecalis* have been shown to increase the villus height and villus height/crypt depth in the jejunum and protect the intestinal villus morphology (Jayaraman et al., 2013). The above results showed that all three probiotics and the complex probiotics were effective in protecting the integrity of the small intestine and promoting the development of intestinal villi. And the complex probiotic treatment was more effective in protecting intestinal integrity.

*Clostridium perfringens* type C is usually infected in the small intestine and mainly affects the epithelium of the jejunum and ileum. In the analysis of the results of the morphology of the small intestine in mice, we found that probiotic treatment was significant in improving the villi height and crypt depth in the ileum. Considering that probiotics may affect the intestinal mucosal barrier and inflammatory response mainly in mouse ileum, we further investigated the tight junctions and immune signaling-related pathways in ileum.

The intestinal physical barrier serves as the first line of defense against the external environment, preventing macromolecules, such as bacteria and toxins, from entering the circulation in the intestinal lumen (Wang K. et al., 2019). The intestinal physical barrier consists of intestinal epithelial cells and tight junctions between cells, and mainly includes proteins such as Muc2, Claudin-1, and Occludin (Cui et al., 2019). Muc2 protein, as the main component of intestinal mucus, can be increased by probiotic treatment (González-Mariscal et al., 2003). In this study, the expression of *Muc2* gene in mouse ileum was downregulated after infection with *C. perfringens* type C and increased after treatment with engramycin or probiotics. Complex probiotic treatment more significantly upregulated the expression of this gene in all treatments in this trial. In addition to mucoprotein, Claudin-1, and Occludin proteins are the backbones that mainly constitute the tightly linked chains that reduce the uptake of toxins by the organism during pathogenic bacterial infections (Phillippi et al., 2022). *Lactobacillus acidophilus* has been shown to increase the expression of Occludin protein for resistance to disruption of the intestinal epithelial barrier by *Escherichia coli* (Mehdizadeh Gohari et al., 2019). In the present study, *C. perfringens* infection decreased the mRNA expression levels of *Claudin-1* and *Occludin* genes in the ileum. Both engramycin and three probiotic treatments increased the mRNA expression level of Claudin-1 gene. And *B. subtilis* BS1-ql and complex probiotics enhanced intestinal tight junctions and reduced infection by upregulating the mRNA expression level of *Occludin* gene. In addition, the changes of tight junctions, adhesive junctions and desmosomes in ileal tissue were observed by Transmission Electron Microscopy. The results showed that the tight junctions between cells were clearly visible in the ileum of NC mice, and from the top of the cells downward, tight junctions, adhesive junctions and desmosomes were visible in order. The gap of tight junctions was widened, the gap of adherent junctions was loosened, and the gap at the adherent band was widened after infection with *C. perfringens*. Tight intercellular junctions at the upper cellular level were clearly visible with engramycin and the three probiotic treatments, and loose local cellular junctions at the middle and lower cellular levels in some cells, but they were reduced compared with the CPC group. The above data suggest that the three probiotics and the complex probiotic enhance the intestinal mucus barrier and tight junctions against the infection caused by *C. perfringens* bacteria by inducing the expression of *Muc2*, *Claudin-1*, and *Occludin* genes. And there may be a synergistic effect between probiotics within the complex probiotic, and the therapeutic effect is better than single probiotic and engramycin treatment.

The intestinal immune barrier is critical in preventing intestinal bacterial infections and maintaining intestinal homeostasis (Resta-Lenert and Barrett, 2003), and Toll-like receptors, the innate immune receptors of the intestine, activate downstream immune pathways by recognizing and binding microbial components (Miranda-Ribera et al., 2019). *Lactobacillus rhamnosus* R0011 and *Lactobacillus acidophilus* R0052 reduced TLR4 expression in mice with alcoholic liver disease (Chassaing et al., 2014). In ulcerative colitis, probiotics reduced the inflammatory factor TNF-α by inhibiting TLR4 expression in colonic tissue (Hong et al., 2015). *Saccharomyces boulardii* CNCM I-745 modulates the intestinal inflammatory response by controlling TLR2 and TLR4, regulating the levels of pro-inflammatory cytokines, and further down-regulating the expression of NF-κB and MAPK signaling pathways (Yang et al., 2013). In the present experiment, *C. perfringens* infection caused an increase in TLR2 mRNA expression in mouse ileal tissue, which further affected the increased gene expression of downstream junction proteins MyD88, IRAK1, TRAF6, and P65, increased pro-inflammatory factor, upregulated MAPK3 and JNK mRNA expression, and participated in the regulation of inflammatory response. Treatment with engramycin and three probiotics and complex probiotics reduced the levels of pro-inflammatory factors (IL-1β, IL-6, TNF-α, and IFN-γ) in the hosts to different degrees. Moreover, the three probiotics and the complex probiotic used in this experiment increased the level of sIgA in the intestine of mice. This result is consistent with the results that probiotics significantly enhanced sIgA and improved canine immunity (Justino et al., 2020), and *Lactobacillus casei* increased the level of secretion in the host jejunum (Wang Y. et al., 2019; Xu et al., 2019). And the effect of the complex probiotic on increasing intestinal sIgA was more significant than that of the single probiotic. sIgA is the main immune factor in the intestine (Ding et al., 2021), blocking the adhesion of antigens such as bacteria, toxins and viruses to the intestinal mucosa and acting as a scavenger of antigens. Treatment with engramycin, three probiotics and their combination probiotic significantly reduced the expression of TLR2 receptor mRNA and significantly downregulated the expression of MyD88, IRAK1, TRAF6 and P65. Differently, the mRNA expression of MAPK3 and JNK were significantly down-regulated by engramycin treatment, while the three probiotics and the complex probiotics did not significantly regulate the expression of MAPK3 and JNK. This may be due to the fact that JNK signaling pathway can also be activated by TNF-α and IL-1β (Davis, 2000). Therefore, Probiotics can regulate the host inflammatory response by affecting the activation of NF-κB and MAPK signaling pathways and the secretion of pro-inflammatory cytokines.

## 5. Conclusion

The results of this study showed that three strains of Tibetan sheep-derived probiotics and probiotic complexes had significant effects in regulating intestinal barrier function and immunity after *C. perfringens* type C infection in mice. After treatment with the three strains of probiotics and the complex probiotics, they could effectively protect the intestinal mucosa, enhance the intestinal barrier function, and affect the TLR2/NF-κB and TLR2/MAPK immune signaling pathways to modulate the inflammatory response of the host. And the complex probiotics showed better regulatory effects than single probiotics and engramycin. This study demonstrated the reliability and effectiveness of different probiotics and complex probiotics in intervening *C. perfringens* infection. Accordingly, *E. faecalis* EF1-mh, *B. subtilis* BS1-ql, *L. sakei* LS-ql and complex probiotics of Tibetan sheep origin may be alternatives to antibiotics for the prevention of *C. perfringens* infections.

## Data availability statement

The original contributions presented in this study are included in the article/Supplementary material, further inquiries can be directed to the corresponding author/s.

## Ethics statement

The animal study was reviewed and approved by the Ethics Committee of Qinghai University (SL-2022010).

## Author contributions

XH: methodology, formal analysis, and writing—original draft. GY: conceptualization and writing—review and editing. SX, XC, and ZG: resources. XH: supervision. All authors contributed to the article and approved the submitted version.
