# Supplementary material for: Effects of three different probiotics of Tibetan sheep origin and their complex probiotics on intestinal damage, immunity, and immune signaling pathways of mice infected with Clostridium perfringens type C
Source: Front Microbiol. 2023 Apr 17;14:1177232. doi: 10.3389/fmicb.2023.1177232 (PMC10149710; doi:10.3389/fmicb.2023.1177232)
Supplement: Supplementary file 1 [file Table_1.DOCX]

Supplementary Material

Effects of three different probiotics of Tibetan sheep origin and their complex probiotics on intestinal damage, immunity and immune signaling pathways of mice infected with *Clostridium perfringens* type C

Xi He^1,2^, Shuqin Xu^1,2^, Xiaohui Chen^1,2^, Xiaolong He^1,2^, Zifeng Gong^1,2^, Guisheng Ye^1,2*^

*** Correspondence:** Guisheng Ye: qhxjygs@163.com

# Supplementary Table

Table S1 Abbreviations and Nomenclature

| Abbreviations | Full Name |
| --- | --- |
| NF-κB | Nuclear Factor Kappa-B |
| MAPK | Mitogen-activated Protein Kinase |
| AP-1 | Activating Protein-1 |
| LB | Luria-Bertani |
| PBS | Phosphate Buffered Saline |
| ELISA | Enzyme Linked Immunosorbent Assay |
| RT-qPCR | Real-time Quantitative Polymerase Chain Reaction |
| SD | Standard Deviation |
| MUC2 | Mucoprotein2 |
| Tlr2 | Toll Like Receptor 2 |
| MyD88 | Myeloid Differentiation Factor 88 |
| IRAK1 | Interleukin 1 Receptor Associated Kinase 1 |
| TRAF6 | Tumor Necrosis Factor Receptor-associated Factor 6 |
| MAPK3 | Mitogen-Activated Protein Kinase 3 |
| JNK | c-Jun N-terminal Kinase |
| DSS | Dextran Sulfate Sodium Salt |
| TLR4 | Toll-like Receptor 4 |

Table S2 List of groups

| Group | Processing Description |
| --- | --- |
| NC | Negative Control |
| CPM | *C. perfringens* Infection Model |
| CPC | *C. perfringens* Infection Positive Control |
| EDC | *C. perfringens* + Engramycin |
| EF | *C. perfringens* + *E. faecalis* EF1-mh |
| BS | *C. perfringens* + B. subtilis BS1-ql |
| LS | *C. perfringens* + *L. sake* SK-ql |
| MC | *C. perfringens* + Multistrain Combination |

Table S3 Primer sequences used for RT-qPCR

| Gene name | Primer sequence (5′-3′) | Product size |
| --- | --- | --- |
| *β-actin* | F: AGGGAAATCGTGCGTGACAT | 149 bp |
|  | R: AACCGCTCGTTGCCAATAGT |  |
| *Claudin-1* | F: GGCTTCTCTGGGATGGATCG | 128 bp |
|  | R: AAACGCAGGACATCCACAGT |  |
| *Occludin* | F: CCTCGGTACAGCAGCAATGG | 123 bp |
|  | R: CCCACCTGTCGTGTAGTCTG |  |
| *MUC2* | F: GACCCAGGAAGTACAGATCAAG | 106 bp |
|  | R: CTCCAGCCCATACTTCTTGTAG |  |
| *IL-6* | F: GAGACTTCCATCCAGTTGCCT | 84 bp |
|  | R: AGTCTCCTCTCCGGACTTGT |  |
| *TNF-α* | F: GCCAACGGCATGGATCTCAA | 139 bp |
|  | R: TAGCAAATCGGCTGACGGTG |  |
| *Tlr2* | F: GGACTTCGTTCCGGGCAAAT | 118 bp |
|  | R: TTCGTACTTGCACCACTCGC |  |
| *MyD88* | F: CCTAGGACAAACGCCGGAAC | 129 bp |
|  | R: CGGTCGGACACACACAACTT |  |
| *MAPK3* | F: TATGACCACGTGCGCAAGAC | 119 bp |
|  | R: TTCTCATGGCGGAATCGCAG |  |
| *IRAK1* | F: CAGAGAATCAAGTGTGAGGAGTAG | 104 bp |
|  | R: TGATCTGTGGTGGTTCTGATG |  |
| *TRAF6* | F: CAGCAGTGTAACGGGATCTAC | 109 bp |
|  | R: CTGTGTAGAATCCAGGGCTATG |  |
| *P65* | F: GCATGCGATTCCGCTATAAATG | 123 bp |
|  | R: GTCCTGTGTAGCCATTGATCTT |  |
| *JNK* | F: GATGAGAGGGAGCACACAATAG | 143 bp |
|  | R: GGATGCTGAGAGCCATTGAT |  |
